# Effect of molar intrusion with temporary anchorage devices in patients with anterior open bite: a systematic review

**DOI:** 10.1186/s40510-016-0122-4

**Published:** 2016-03-23

**Authors:** Ahmad Saleem Alsafadi, Mohannad M. Alabdullah, Humam Saltaji, Anas Abdo, Mohamed Youssef

**Affiliations:** Department of Orthodontics, Faculty of Dentistry, Damascus University, Damascus, Syria; Orthodontic Graduate Clinic, Faculty of Medicine and Dentistry, University of Alberta, Edmonton, Canada; Faculty of Dentistry, Damascus University, Damascus, Syria

**Keywords:** Systematic review, Skeletal open bite, Molar intrusion

## Abstract

**Objective:**

The objective of the study is to assess the effect of molar intrusion with temporary anchorage devices on the vertical facial morphology and mandibular rotation during open bite treatment in the permanent dentition.

**Methods:**

We performed a systematic review of the published data in seven electronic databases up to September 2015. We considered studies for inclusion if they were examining the effects of posterior teeth intrusion on the vertical facial morphology with open bite malocclusion in the permanent dentition. Study selection, risk of bias assessment, and data-extraction were performed in duplicate. Meta-analysis was not possible due to dissimilarity and heterogeneity among the included studies.

**Results:**

Out of the 42 articles that met the initial eligibility criteria, 12 studies were finally selected. Low level of scientific evidence was identified after risk of bias assessment of the included studies with no relevant randomized controlled trial performed. Out of the 12 selected studies, five studies used miniplates and seven studies used miniscrews. Mandibular counterclockwise rotation was found to be between 2.3° and 3.9° in six studies (as sassed by mandibular plane angle, between MeGo or GoGn and SN or FH plane) while it was less than 2° in the remaining studies.

**Conclusions:**

Current weak evidence suggests that molar intrusion with temporary anchorage devices may cause mandibular counterclockwise autorotation. Future well-conducted and clearly reported multicenter randomized controlled trials that include a non-treatment control group are needed to make robust recommendations regarding the amount of mandibular rotation during open bite treatments.

## Review

### Introduction

Open bite malocclusion is considered one of the most difficult orthodontic problems to correct because it appears as a result of the interaction of numerous etiological factors (genetic, dental, skeletal, functional, soft tissue, and habit) that contribute to its development [1]. An open bite can occur unilaterally or bilaterally in the buccal segments; it is particularly seen in the anterior teeth. Generally, different features have been found to be associated with the skeletal anterior open bite distinguishing it from other types of malocclusion including increased lower face height, short posterior face height, [2] increased gonial and mandibular plane angles, [3] and increased maxillary molar dentoalveolar height [4]. Several reports have found correlations between orofacial muscle activity and vertical facial morphology [5–8]. These studies showed positive relationships between anterior open bite and weak musculature.

Various therapeutic approaches have been proposed for the treatment of an anterior open bite. These approaches vary depending on the causative factors and involve myotherapy, preventive treatment, functional therapy, orthognathic surgery, and orthodontic treatment using anterior teeth extrusion or posterior teeth intrusion [9]. Among the non-surgical orthodontic treatment methods are the temporary anchorage devices (TADs) including miniplates [10] and miniscrew or micro-screw implants [9, 11]. Extrusion of anterior teeth is another alternative approach for open bite management, but it must take into consideration the smile esthetic [12]. Extrusion, however, is a less stable treatment than intrusion. The intrusion of posterior teeth with temporary anchorage devices was suggested to lead to decreased lower facial height by a counterclockwise rotation of the mandible; this may resemble the orthognathic surgery outcomes for any open bite patients [10].

Molar intrusion is recommended in open bite patients who usually have increased molar height [13]. While many reports indicated that increased molar height is one of the common findings in individuals with skeletal open bite [14], others do not support those findings [15, 16]. In order to evaluate the results of molar intrusion in the treatment of open bite malocclusion, it is necessary to recognize the effect of the posterior teeth intrusion on the mandibular rotation and facial morphology.

Many reports evaluated the effect of open bite treatment during mixed dentition stage [17] and [18]. A recent systematic review examined open bite treatment modalities in children found no consistent findings regarding the most effective treatment modality in growing patients with open bite malocclusion [19]. However, no comprehensive review was conducted to examine the effects of posterior teeth intrusion on vertical facial morphology in non-growing patients. Therefore, the goal of the current report is to systematically review the effect of molar intrusion with temporary anchorage devices on the vertical facial morphology and mandibular rotation during open bite treatment in the permanent dentition stage.

### Material and methods

This systematic review was reported according to the principles of the PRISMA statement for reporting systematic reviews of the health sciences [20].

#### Search strategy

Comprehensive electronic searches up to September 30, 2015, were conducted in the following databases: PubMed, Embase, Cochrane Database of Systematic Reviews, Cochrane Central Register of Controlled Trials, Ovid, Scopus, and Web of science. The literature searches used the following Medical Subject Headings (MeSH) terms: “molar intrusion,” “posterior teeth intrusion,” and “anterior open bite” which were crossed with the following terms “mandibular autorotation” and “facial morphology.” In addition, the following journals were searched individually to find out any missing articles: *The Angle Orthodontists*, *American Journal of Orthodontics and Dentofacial Orthopedics*, *European Journal of Orthodontics*, *Korean journal of orthodontics*, *and Journal of Orofacial Orthopedics*. No restrictions were applied regarding date of publication, language, or status during database searches. The search strategy for PubMed can be found in Table 1.

**Table 1:** Search strategy in PubMed

#1 molar intrusion OR posterior teeth intrusion OR anterior open bite
#2 facial morphology OR mandibular autorotation
#3 #1 AND #2

#### Focused question

What is the effect of molar intrusion with temporary anchorage devices on the vertical facial morphology and mandibular rotation in the permanent dentition stage during open bite treatment?

#### Selection criteria (PICO question: population, intervention, comparison, outcome)

The following eligibility criteria were used to determine eligible reports for this systematic review:

- Population: Adolescent and adult patients with anterior open bite malocclusion. Studies examining patients with craniofacial anomalies or syndromes, cleft lip and/or palate, surgically assisted treatment, and patients in the mixed dentition stage were excluded from the review. Only human studies were included without consideration of gender.
- Intervention: Patients undergoing orthodontic treatment for open bite correction by posterior teeth intrusion in the upper and/or lower arch by using temporary anchorage devices.
- Comparison: A temporary anchorage devices technique for posterior segment intrusion vs. control or another equivalent intervention, or before and after treatment.
- Outcomes: Angular and linear measurements used to assess the vertical changes of the mandible: overbite and maxillary and mandibular plane angle (MMA); mandibular plane angle (MPA) between Go-Gn or Me-Go and reference plane (FH or SN); lower anterior facial height (LAFH), the distance between anterior nasal spine ANS and point Me; Jarabak ratio, the ratio between posterior facial height (PFH) and anterior facial height (AFH); *Y*-axis angle, the angle between Sell-Nasion (SN) and Sell-Gnathion (S-Gn); the distance between lower first molar (L1) and mandibular plane; and the distance between upper first molar U6 and the reference plane, either palatal plane or horizontal plane.
- Study design: Randomized and non-randomized controlled clinical trials, clinical trials (prospective and retrospective), and case series studies. Excluded articles included case reports with ≤8 subjects, animal studies, review articles, abstracts, and discussions.

#### Study selection

The titles and abstracts of all articles obtained through the electronic searches were screened independently by three reviewers (ASA, MA, and AA). Since it cannot rely on abstracts to get enough information about the results of posterior teeth intrusion on the facial structure, no attempt at this stage was made to identify studies that did not mention the effect of molar intrusion on facial morphology and mandibular rotation. After obtaining a sufficient number of abstracts, full articles were retrieved for the final selection process. The reference list of the articles that have been retrieved was checked out for additional studies that may have been missed in the initial searches. A consensus was reached among the assessors about the articles that met the eligibility criteria.

#### Data collection and analysis

Data was extracted in duplicate by two reviewers (ASA and MA) on the following items: year of publication, study design, materials, method measurements, age, sample size, treatment period, force applied, amount of reduction of open bite, mandibular rotation obtained, improvement of facial morphology gained, side effects, and author’s conclusions, among others.

#### Risk of bias in individual studies

The risk of bias of included trials was assessed using the methodological index for non-randomized trials (MINORS) tool (Table 2) [21]. Two reviewers (ASA and MA) performed the evaluations, and in cases of disagreement, consensus was reached after discussion. Methodological quality was done for each article without blinding to the authors.

**Table 2:** Risk of bias assessment of included studies

Quality item	Sugawara et al. 2002 [22]	Deguchi et al. 2011 [23]	Buschang et al. 2011 [24]	Akan et al. 2013 [25]	Xun et al. 2007 [26]	Erverdi et al. 2004 [27]	Erverdi et al. 2007 [28]	Scheffler et al. 2014 [29]	Foot et al. 2014 [30]	kuroda et al. 2007 [10]	Hart et al. 2015 [32]	Lee and Park 2008. [31]
1. A clear stated aim	1	2	1	2	2	2	2	1	2	2	2	2
2. Inclusion of consecutive patients	0	2	2	0	0	0	0	2	1	0	2	0
3. Prospective collection of data	0	1	2	1	1	0	1	0	2	1	1	1
4. Endpoints appropriate to the aim of the study	2	2	1	2	2	2	1	2	2	2	2	1
5. Unbiased assessment of the study end point	0	0	0	0	0	0	0	0	0	0	0	0
6. Follow-up period appropriate	2	2	0	0	0	0	1	2	0	0	0	2
7. Loss to follow-up less than 5 %	2	2	0	0	0	0	0	0	0	0	0	2
8. Prospective calculation of the study size	0	0	0	0	0	0	0	0	0	0	0	0
Additional criteria in the case of comparative study												
9. An adequate control group	2	2	–	–	–	–	–	–	–	2	–	–
10. Contemporary groups	0	2	–	–	–	–	–	–	–	2	–	–
11. Baseline equivalence of groups	0	1	–	–	–	–	–	–	–	1	–	–
12. Adequate statistical analyses	2	2	0	2	2	1	2	2	2	2	2	2
Total	11	18	6	7	7	5	7	9	9	12	9	10

#### Data synthesis

We planned to perform a meta-analysis if both quality and quantity of the information retrieved from the finally selected studies justified a meaningful statistical combination.

### Results

**Fig 1:**
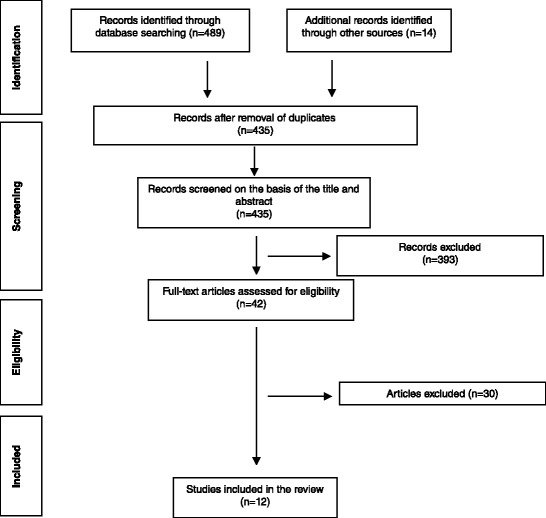
Flow diagram of the literature search

Among 503 articles retrieved as a result of the initial searching process, 393 articles were excluded according to the information provided in their titles and abstracts, while 68 articles were excluded as they were duplicate articles. Consequently, 42 studies remained for the eligibility process, and eventually, only 12 studies fulfilled the inclusion criteria [10, 22–32]. One article by Hart et al. [32] was identified after the date of our search. Table 3 shows the study design and characteristics of the final selected articles. After searching manually within references of the approved articles, it was found that all related studies were included in the initial electronic search process. Figure 1 shows the scheme of article selection and the number of articles accepted and excluded. The complete list of excluded articles and reasons for exclusion are available upon request.

**Table 3:** Characteristics of studies included in systematic review

Author and year of publication	Sample size and age	Comparison	Study design	Method measurement	Study materials	Treatment time	Force applied	Reduction of open bite	Effect on mandibular autorotation	Effect on facial morphology	Outcomes assessed	Side effects	Author’s conclusion
Sugawara et al. (2002) [22]	SAS (9) (13.3 to 28.9 yrs)	Pre- and post-, one group only (miniplates)	R, CS	Lateral cephalometric analysis, panoramic analysis, dental cast analysis	SAS miniplate (L shaped) in lower molars only	SAS 14.9 mo (9 to 22 mo) follow-up 12 mo	Not declared	↑ overbite by 4.9 mm ↓ overbite 0.9 mm after 1 yrs follow-up	Counterclockwise rotation ↓ FH/MP by 1.3° ↑ FH/MP by 0.4° after 1 yrs follow-up	↓ALFH, ↓ interlabial gap and improve AP jaw relation. Stable profile after 1 yrs follow-up.	Overbite MP/FH LAFH U6-PP L6-MP	27.2 % rate of relapse in the 1st molars and 30.3 % in the 2nd molars.	SAS is effective for open bite treatment; overcorrection is necessary.
Deguchi et al. (2011) [23]	G1: non-IA (15) 22.9 yrs; G2: IA (15) 25.7 yrs	Two-group comparison (non-implant anterior elastics and HPHG with MEAW vs. implant group)	R, L, CT	Lateral cephalometric analysis, cast analysis PAR and DI scores	G1: non-implant group (HPHG, MEAW, or accentuated COS with elastics); G2: implant group (sectional wire in upper and lower posterior segment)	G1: non- IA 1–3 yrs; G2: IA 1–3 yrs 2 yrs follow-up	Not declared	G1: ↑ OB by 6.5 mm ↓ 0.5 mm after 2 yrs G2: ↑ OB by 6.2 mm ↓ 0.8 mm after 2 yrs	G1: ↑ MP/SN by 2.7° clockwise rotation, ↑ 0.3 mm after 2 yrs G2: ↓ MP/SN by 3.6° counterclockwise rotation, ↑1.6° after 2 yrs	G1: ↑ AFH and ↓facial convexity, and ↓lips protrusion G2: ↓ AFH, more ↓ in facial convexity, and ↓ lips protrusion Disappearance of incompetent lips.	Overbite MeGo/SN LAFH U6-PP L6-MP	Less molar torque control, extrusion of lower molars. 1 patient in G1 and 2 patients in G2 showed relapse after 2 yrs.	G2 showed more relapse than G1, overcorrection and myofunctional therapy is recommended. Keep screws longer time or use retainer with occlusal stops in mandible.
Buschang et al. (2011) [24]	MSIs (9) 13.2 yrs	Pre- and post-, one group only (miniscrews)	P, CS	Lateral cephalometric analysis	MSIs miniscrew implants in upper posterior segment (with RPE) + buccal miniscrews in lower molars	1.9 yrs (1.4 to 2.5 yrs)	150 g per side	Not declared	↓ MPA by 3.9° counterclockwise rotation	Chin moved forward by 2.4 mm ↑ SNB, and ↓ facial convexity	MPA	Lower molar eruption. No stability information.	Using MSIs for intrusion is effective, not painful or uncomfortable.
Akan et al. (2013) [25]	Miniplate (19) 17.7 yrs	Pre- and post-, one group only (miniplate + acrylic plate)	P, CS	Lateral cephalometric analysis PA radiograph EMG and EVG recording	Surgical miniplates in upper posterior segment only	6.8 mo	400 g per side	↑Overbite by 4.79 mm	↓ Go Gn/SN by 3.79° counterclockwise rotation	↑ SNB, ↓ LAFH, ↓ AFH, ↓ facial convexity, and ↑upper lip/ E plane	Overbite MP/FH GoGn/SN LAFH U6-HL L6-MP	Insignificance tipping molars buccally. No stability information.	Miniplate is successful Tx and has no effect on TMJ and masticatory muscles activity.
Xun et al. 2007 [26]	Miniscrews (12) 18.7 yrs	Pre- and post-, one group only (miniscrews)	R, CS	Lateral cephalometric analysis	Midpalatal miniscrew in upper arch + buccal miniscrews in lower molars	6.8 mo	150 g per side	↑ overbite by 4.2 mm	↓Me Go/ SN by 2.3° counterclockwise rotation	↓ AFH, ↓ LAFH, and ↓ Ns-Sn-Pos improvement of convex profile.	Overbite MMA MeGo/SN LAFH U6-PP L6-MP	No stability information.	Miniscrew provide stable, simple, and less invasive anchorage.
Erverdi et al. (2004) [27]	Miniplate (10) 17-23 yrs	Pre- and post-, one group only (miniplate)	P, CS	Lateral cephalometric analysis PA radiograph analysis	Miniplate (I shaped) sectional wire in upper posterior segment only, (ext of upper 1st premolar in 6 P)	5.1 mo	Not declared	↑ overbite by 3.7 mm	↓ Go Gn / SN by 1.7° counterclockwise rotation	↓ AFH, ↑ glabella-SN-pogonion. Improve smile and profile.	Overbite MMA GoGn/SN U6-PP L6-MP	Upper molars tipped buccally slightly, tissue inflammation and irritation of cheeks. No stability information.	Minimal invasive and simple technique for intrusion, long-term follow-up required.
Erverdi et al. (2007) [28]	Miniplate (11) 19.5 yrs	Pre- and post-, one group only (miniplate + acrylic plate)	P, L, CS	Lateral cephalometric analysis	Miniplate (I shaped) In upper posterior segment only	9.6 mo 1 yrs follow-up	400 g per side	↑ overbite by 5.1 mm	↓ Go Gn / SN by 3.0° counterclockwise rotation	↓ LAFH, ↑ SNB	Overbite GoGn/SN LAFH U6-PP	Minor edema, pain, and extrusion of lower molars. No relapse regarding rotation at 1 yrs follow-up, few cases showed extrusion 1 mm of upper molars.	Therapy is effective. Longer follow-up required with large number of patients.
Scheffler et al. (2014) [29]	Miniplates & miniscrews (30) (12.7 to 48.1 yrs) 24.1 yrs	Pre- and post-, one group only (miniplate &miniscrew + acrylic plate)	R, L, CS	Lateral cephalometric analysis	Miniscrews (16 P) or miniplates (14 P) in upper posterior segment only	3.6–9.6 mo for intrusion 6–33 mo total tx time	150 g per side	↑ Overbite by 2.2 mm	↓ Go Gn/SN by 1.2° counterclockwise rotation ↑ 0.2 at debonding No change at 1 and 2 yrs follow-up	↓ LAFH decrease LAFH by a fraction of a millimeter at 1 and 2 yrs follow-up	Overbite GoGn/SN LAFH U6-PP L6-GoGn	Extrusion of lower molars, elongation of upper and lower incisors. 15 and 22 % of the patients showed relapse (>1 mm) in overbite at 1 and 2 yrs follow-up, respectively.	Control vertical position of mandibular molars. Some intrusion of maxillary canine is needed.
Foot et al. (2014) [14]	SIS (16) (12.2 to 14.3 yrs) 13.1 yrs	Pre- and post-, one group only (miniscrews + acrylic plate)	P, CS	Conebeam + lateral cephalometric analysis	Sydney intrusion spring (SIS) in upper posterior segment only	4.91 mo (2.5 to 7.7 mo)	500 g per side	↑ Overbite by 3 mm	↓ MP/SN by 1.2° counterclockwise rotation	↓ LAFH, ↓ G’SnPo’	Overbite MP/FH MMA GoGn/SN LAFH U6-PP L6-MP	Tissue irritation, difficulty in adaptation and maintaining of appliance. No stability information.	Effective appliance for posterior teeth intrusion with minimal reactivation, and well tolerated by patients.
Kuroda et al. (2007) [10]	G1: miniplate or miniscrew (10) G2: orthogna-thic surgery (13) (16-46 yrs) 21.6 yrs	Two-group comparison (miniplate or miniscrew group vs. orthognathic surgery group)	P, CT	Lateral cephalometric analysis	G1: TADs sectional wire in upper and lower posterior segment G2: LeFort 1 osteotomy and intraoral vertical ramus osteotomy or sagittal split ramus osteotomy	G1: 27.6 mo (19–36 mo) G2: 33.5 mo (20–44 mo)	G1: 150 gm G2: not declared	G1: ↑ overbite by 6.8 mm G2: ↑ overbite by 7 mm	G1: counterclock-wise rotation ↓ FH/MP by 3.3° G2: counterclock-wise rotation ↓ FH/MP by 0.3°	G1: ↓ TAFH & LAFH better morphologic improvement than surgery G2: ↓TAFH, no change in LAFH	Overbite MP/FH LAFH U6-PP L6-MP	Elongation of incisors in orthognathic surgery group. No stability information.	Molar intrusion by skeletal anchorage is simpler and cause more rotation of mandible than surgery.
Hart et al. (2015) [32]	Palatal miniscrews (31) (11.6 to 55.5 yrs) 20.7 yrs	Pre- and post- one group only (palatal miniscrews) (21 adolescent vs. 10 adult patients)	R, CS	Lateral cephalometric analysis	Bilateral perimolar palatal miniscrews (25 p) and midpalatal miniimplants (6 p) in upper arch only (uses TPA + QH to control intermolar width)	1.3 yrs	Not declared	↑ Overbite by 3.8 mm	↓ FH/MP by 1.1° counterclockwise rotation	↓ LAFH, ↓ AFH ↓ PFH decrease in skeletal class II features	Overbite MP/FH LAFH U6-PP U6-BaH L6-MP	Extrusion of lower first and second molars, distal tipping of maxillary 1st molars. No stability information.	Miniscrews provide adequate anchorage for molar intrusion. Adolescent patients showed more favorable mandibular autorotation than adults. mandibular molar positions could be controlled by occlusal coverage with retainer, or full fixed appliance.
Lee HA, and Park YC. (2008) [31]	Miniscrews (11) (18.2 to 31.1 yrs) 23.3 yrs	Pre- and post- one group only (buccal miniscrews)	P, L, CS	Lateral cephalometric analysis	Miniscrews with palatal rigid splint to prevent molar tipping sectional wire in upper posterior segment only.	5.4 mo	Not declared	↑ Overbite by 5.47 mm ↓ 0.99 mm after 17.4 mo retention period	Counterclockwise rotation ↓ Me Go/SN by 2.0° ↑ Me Go/SN 0.18° after 17.4-mo retention period	↓ AFH, pogonion moved 2.17 mm forward. Improve esthetic with muscle adaptation. ↑ AFH by 0.38 mm after 17.4 mo retention period.	Overbite MeGo/SN MP/FH AFH U6-PP	10.36 % relapse rate for molar intrusion, and 18.10 % relapse rate for overbite after 17.4-mo retention period.	Intrusion of maxillary posterior teeth by using miniscrews in adult patients is an effective method for open bite correction with good stability.

*Abbreviation*: *P* prospective, *R* retrospective, *CT* clinical trial, *CS* case series, *L* longitudinal, ↑ increase, ↓ decrease, *ext* extraction, *SAS* skeletal anchorage device, *COS* curve of Spee, *tx* treatment, *RPE* rapid palatal expansion, *PA* posterior-anterior, *mo* months, *yrs* years, *P* patients, *Me* Menton, *Go* Gonion, *Gn* gnathion, *SN* Sella-Nasion, *MP* mandibular plane, *FH* Frakfurt horizontal, *MPA* mandibular plane angle, MMA maxillary mandibular angle, *U6* upper first molar, *PP* palatal plane, *HL* horizontal line, *BaH* basion horizontal plane parallel to FH, *TPA* transpalatal arch, *QH* quad helix

Different measurements have been used to determine the outcomes of the skeletal changes resulting from posterior teeth intrusion during open bite treatment. Table 4 shows linear and angular measurements mentioned in the selected reports, which indicate the amount of mandibular autorotation and the consequent improvement in facial esthetics.

All studies included in our methodological scoring process have low-moderate quality as presenting in Table 2. Randomization, sample size evaluation, and blinding were not mentioned in any studies. Three clinical trials with control groups were found in this review [10, 22, 23]. The criteria used to assess the amount of molar intrusion and mandibular rotation with its effect on facial morphology were stated properly by nine articles [10, 22, 23, 25–27, 29–32], while follow-up length stability was examined in five reports [22, 23, 28, 29, 31].

Out of the 12 selected studies, five studies [10, 22, 25, 27, 28] used miniplates and seven studies [23, 24, 26, 29–32] used miniscrews. The amount of mandibular rotation was more than 2° in six studies [10, 23–26, 28]. The maximum amount of mandibular counterclockwise rotation was found to be 3.9° [24], while the lowest amount was 1.1° as reported by Hart et al. [32] by using miniscrews in the upper arch only. In the Kuroda et al. [10] study, 3.3° mandibular counterclockwise rotation was achieved using temporary anchorage devices compared with the orthognathic surgery. Detailed report of outcome measurements and characteristics for each study are presented in Tables 3 and 4.

**Table 4:** Summary of outcome measurements of the selected studies

Article	Outcomes	Before TX mean (SD)	After TX mean (SD)	Difference mean (SD)	Follow-up changes
Sugawara et al. (2002) [22]	Overbite	−2.8 (1.8)	2.1 (0.8)	4.9 (NA)	1.2 (0.8)
	MP/FH	33.1 (2.1)	31.7 (2.4)	−1.3 (NA)	32.2 (3.0)
	L6-MP	35.7 (4.1)	33.9 (4.1)	−1.8 (NA)	34.2 (4.4)
	U6-PP	24.0 (3.0)	25.0 (2.8)	1.0 (NA)	25.1 (2.5)
	LAFH	76.1 (5.8)	74.6 (6.0)	−1.5 (NA)	75.2 (5.8)
Deguchi et al. (2011) [23]	Overbite	−4.4 (1.2)	1.8 (1.1)	6.2 (1.7)	1.0 (0.9)
	MP/SN	45.8 (6.0)	42.2 (6.7)	−3.6 (2.1)	43.8 (6.5)
	U6-PP	26.9 (3.0)	24.6 (2.5)	−2.3 (1.3)	25.1 (2.8)
	L6-MP	36.0 (2.5)	35.2 (1.9)	−0.8 (1.3)	37.0 (1.9)
	LAFH	74.7 (5.9)	72.2 (5.1)	−2.6 (2.5)	72.2 (5.1)
Buschang et al. (2011) [24]	MPA	NR	NR	−3.9 (1.8)	NA
Akan et al. (2013) [25]	Overbite	−3.2 (1.3)	1.5 (1.3)	4.7 (1.3)	NA
	GoGn/SN	45.5 (7.3)	41.7 (7.2)	−3.7 (1.8)	
	MP/FH	36.3 (7.0)	33.0 (6.9)	−3.2 (1.5)	
	LAFH	57.0 (4.9)	52.8 (4.6)	−4.1 (1.7)	
	U6-HL	74.6 (4.4)	71.3 (4.2)	−3.3 (1.2)	
	L6-MP	32.0 (3.2)	31.2 (3.3)	−0.8 (0.8)	
Xun et al. (2007) [26]	Overbite	−2.2 (0.9)	2.0 (0.3)	4.2 (0.9)	NA
	MMA	36.8 (4.8)	34.3 (4.2)	−2.5 (0.9)	
	MP/SN	45.6 (5.8)	43.3 (5.1)	−2.3 (0.8)	
	LAFH	82.2 (3.8)	80.6 (3.4)	−1.6 (0.9)	
	U6-PP	26.3 (2.2)	24.5 (2.0)	−1.8 (0.7)	
	L6-MP	37.4 (3.4)	36.2 (3.3)	−1.2 (0.8)	
Erverdi et al. (2004) [27]	Overbite	−0.6 (2.2)	3.1 (0.9)	3.7 (2.4)	NA
	MMA	36.9 (5.4)	34.7 (4.5)	−2.2 (1.2)	
	GoGn/SN	46.5 (6.0)	44.8 (6.7)	−1.7 (2.0)	
	U6-PP	26.4 (1.6)	23.8 (2.7)	−2.6 (1.3)	
	L6-MP	32.8 (2.7)	31.7 (3.1)	−0.1 (0.7)	
Erverdi et al. (2007) [28]	Overbite	−4.0 (NA)	1.2 (NA)	5.1 (2.0)	NA
	GoGn/SN	42.5 (NA)	39.5 (NA)	−3.0 (1.5)	
	LAFH	76.5 (NA)	73.6 (NA)	−2.9 (1.3)	
	U6-PP	22.6 (NA)	19.0 (NA)	−3.6 (1.4)	
Scheffler et al. (2014) [29]	Overbite	NR	NR	2.2 (1.6)	−0.4 (1.1)
	GoGn/SN			−1.2 (1.0)	0.0 (0.8)
	LAFH			−1.6 (2.2)	−0.3 (1.4)
	U6-PP			−2.3 (1.4)	0.5 (1.2)
	L6-GoGn			0.6 (1.6)	−0.3 (1.3)
Foot et al. (2014) [30]	Overbite	−2.2 (1.7)	0.8 (1.1)	3.0 (1.5)	NA
	MMA	31.8 (3.8)	30.7 (4.3)	−1.0 (1.3)	
	MP/SN	36.1 (4.9)	34.9 (5.3)	−1.2 (1.3)	
	MP/FH	27.8 (3.5)	26.9 (3.6)	−0.9 (1.2)	
	LAFH	61.8 (4.3)	60.9 (4.6)	−0.9 (1.1)	
	U6-PP	20.6 (2.3)	17.7 (2.4)	−2.9 (0.8)	
	L6-MP	27.9 (2.9)	28.0 (2.9)	0.1 (0.4)	
Kuroda et al. (2007) [10]	Overbite	−5.2 (1.8)	1.5 (0.6)	6.8 (1.7)	NA
	MP/FH	38.8 (6.4)	35.5 (6.9)	−3.3 (1.5)	
	LAFH	78.6 (5.5)	75.0 (4.7)	−3.6 (1.8)	
	U6-PP	28.7 (2.8)	26.4 (2.4)	−2.3 (2.0)	
	L6-MP	38.0 (1.8)	36.7 (1.6)	−1.3 (1.2)	
Hart et al. (2015) [32]	Overbite	−3.0 (1.9)	0.8 (1.4)	3.8 (0.9)	NA
	MP/FH	32.4 (6.3)	31.3 (6.9)	−1.1 (0.09)	
	LAFH	73.3 (7.4)	71.8 (7.1)	−1.5 (0.03)	
	U6-PP	23.7 (3.3)	21.4 (3.2)	−2.3 (0.06)	
	U6-BaH	27.4 (3.7)	25.7 (3.7)	−1.7 (0.10)	
	L6-MP	30.4 (3.1)	31.5 (3.3)	1.1 (0.05)	
Lee and Park. (2008) [31]	Overbite	−3.7 (1.7)	1.7 (0.7)	5.4 (1.8)	0.7 (0.7)
	MeGo/SN	44.9 (3.9)	42.9 (4.5)	−2.0 (1.7)	43.0 (4.8)
	MP/FH	37.9 (3.1)	35.0 (3.6)	−2.8 (2.0)	35.9 (3.8)
	AFH	133.4 (5.4)	130.8 (5.7)	−2.6 (1.9)	131.1 (5.4)
	U6-PP	26.7 (1.2)	24.5 (1.7)	−2.2 (1.7)	24.7 (1.6)

*Abbreviation*: *SD* standard deviation, *NR* not reported, *NA* not available

Both dissimilarity and heterogeneity was found in the outcome measures, after analyzing data in the related studies. As a result, meta-analysis was not possible for this systematic review.

### Discussion

Molar intrusion is one of the valid approaches used for open bite treatment. While true molar intrusion was quantified clinically in a previous review [33], no systematic review was conducted to examine the effect of molar intrusion on the facial morphology and mandibular autorotation in the permanent dentition.

Despite progress in orthodontic treatment techniques, open bite treatment still represents a challenge for orthodontists. While orthognathic surgery is considered the gold standard for skeletal open bite treatment to achieve the optimal esthetic and occlusal result [34], non-surgical orthodontic treatment for these cases has become more common since it is less expensive and more acceptable for patients. Early treatment of open bite during mixed dentition has been supported by many authors [35], as it represents the importance of modification of habits or reducing the need for orthognathic surgery after puberty. Therefore, many reports were discussing open bite treatment in the younger age group. Furthermore, difficulty in obtaining open bite patients has made most reports in our electronic search are case reports.

Our systematic review identified five studies that used miniplates for molar intrusion, three of which [25, 27, 28] applied them in the upper jaw, one study in the lower jaw [22], and one study applied TADs in both arches [10]. The applied force was 400 g on each side through the intrusion for a period ranged between 5.1 months [27] and 14.9 months [22]. Two reports [22, 27] did not mention the amount of applied force. Mandibular counterclockwise rotation was a common result in the miniplates groups.

Regarding posterior teeth intrusion in one arch, Akan et al. [25] and Erverdi et al. [28] showed 3.7° and 3.00° of mandibular counterclockwise rotation, respectively, which is higher than the value of counterclockwise rotation mentioned in the remaining two studies [22, 27], probably because of adding a posterior bite block so as to help for posterior teeth intrusion through stimulating muscular response. The amount of inter-labial gap and facial convexity was decreased after mandibular counterclockwise rotation [22, 25].

Through using miniscrews for intrusion of the lower mandibular molars and upper posterior teeth, the autorotation has increased to become 3.9°, which is the maximum among approved articles [24]. According to Foot et al. [30], only 1.2° of mandibular rotation was achieved; this might be explained by using a subject group with an average amount of pretreatment open bites of 2.6 mm that required lesser amount of intrusion. Even when using a posterior occlusal splint in one group, Scheffler et al. [29] reported similar amount of mandibular rotation (1.2°). This is probably because canines remain in contact after removal of the splint; this causes a reduced degree of mandibular autorotation and correction of overbite. Hart et al. [32] reported the lowest amount of mandibular rotation (1.1°); this might be interpreted by continuous eruption of lower first and second molars. Thus, the amount of mandibular rotation would be limited as the mandibular molars eruption compensating the amount of mandibular counterclockwise rotation. These changes are more significant in adolescent patients. Moreover, facial convexity and lip protrusion decreased significantly after posterior teeth intrusion by miniscrews [23, 24, 26, 31]. Miniscrews were placed in the upper and lower arch for molar intrusion; the applied force ranged between 150 g on each side [24, 26, 29] and 500 g per side [30]. Deguchi et al. [23] suggested that conventional treatment without miniscrews could not result in improvement in facial profile as if the miniscrews were applied for intrusion. The multiloop edgewise archwire (MEAW) technique had been the most common treatment before invention of TADs. Deguchi et al. [23] reported that using the MEAW technique improved overbite without achieving mandibular autorotation or intrusion in the upper or lower molars. The increase in overbite was because of anterior intermaxillary elastics.

The magnitude of the mandibular autorotation after molar intrusion was dependent on a set of interrelated factors including amount of intrusive force, duration of intrusion, and place of intrusion in upper or lower arch. With respect to the amount of intrusive force, it has been reported that using 400 g per segment for posterior tooth intrusion using miniplates will lead to 2° to 4° of mandibular counterclockwise rotation measured between mandibular plane (GoGn) and SN plane [25, 28]. Similar to the result of the upper and lower molar intrusion simultaneously by measuring the angle between mandibular plane (MeGo) and SN or FH, after application of a 150 g to each side by means of miniscrews [24, 26] or with combination with miniplates [10], the longest period for molar intrusion was achieved by applying miniplates in lower arch only for intrusion [22]. However, the intrusion time was ranged between 5 and 10 months [25–29, 31]. Increasing both the amount of intrusion force and miniscrews number will lead to decrease in the duration of intrusion to 4.9 months [30]. Generally, intrusion of the upper and lower molars simultaneously will increase the amount of the mandibular rotation and correction of the open bite [10, 23]. It seems that using an acrylic plate along with the miniplate for upper molar intrusion will lead to intrusion of the lower molars [25, 27]; this was not confirmed in other studies [29] and [30]. Although the amount of intrusion in the lower arch was statistically non-significant, it could assist in the mandibular counterclockwise rotation. Intrusion of upper posterior teeth by means of temporary anchorage devices without acrylic plate will lead to overeruption of lower molars [32]. Occlusal coverage with a vacuum retainer or full fixed appliances with lower molar engagement could mitigate the effect of uncontrolled eruption of mandibular molars. 

Comparing pre- and post-treatment measurements in the consecutive radiographs might not be a valid method to evaluate the linear and angular changes of the facial structures. Radiograph superimposition is considered an accurate method for facial changes assessment as a result of growth or orthodontic treatment and that after registration on the stable reference areas [36]. Three reports [10, 24, 27] used cephalometric superimposition without details of the reference structures. Anterior contour of the chin, inner contour of the cortical plates at the inferior border of the symphasis, and contours of the mandibular canal are well documented as stable structures for serial superimposition [37]. Mandibular autorotation has been assessed by the angular position of the mandibular plane (MP) relative to the FH plane [10, 22, 31, 32] or to the cranial base (SN) [23, 25–30]. However, none of the studies measured the Jarabak ratio and *Y*-axis angle. Others used MMA to evaluate maxillary or mandibular rotation [26, 27, 30].

Improvement of facial esthetics by decreasing LAFH was mentioned in most studies except those conducted by Buschang et al. [24] and Erverdi et al. [27]. Soft tissue changes must be quantified carefully in order to assess proper changes in them. The use of 3D is a promising method to evaluate soft tissue changes during open bite treatment provided that they are compared with the control group and obtain an accurate measurement.

#### Limitations

There are no randomized clinical trials performed focused on the open bite treatment using temporary anchorage devices. Presence of randomization is an important issue to consider when determining the best treatment modality for posterior teeth intrusion. It is clinically important to investigate the amount of mandibular rotation during open bite treatment by means of miniscrews and/or miniplates in comparison with other therapeutic treatment options (such as MEAW, premolars extraction, high-pull headgear, and orthognathic surgery), as well as evaluation of the long-term stability of posterior teeth intrusion by different techniques. The drawbacks in most of the articles such as absence of untreated control groups, short follow-up period, small sample size, and presence of confounding factors should be avoided in future studies so as to reach a more accurate conclusion concerning open bite treatment.

## Conclusions

Current available evidence suggests that that posterior teeth intrusion in the permanent dentition stage using TADs might cause mandibular counterclockwise rotation and improve facial esthetics. Miniscrews showed 2.3° to 3.9° of mandibular counterclockwise rotation (as sassed by mandibular plane angle, between MeGo or GoGn and SNFH plane) when an intrusive force applied to both upper and lower molars, which is almost similar to what was observed after application of the high intrusive force in the upper posterior segment only by means of miniplate and acrylic bite block. Absence of a standardized method of intrusion, outcome measurements, and differences in the protocols followed for molar intrusion (in one arch or both arches) have led to concluding weak clinical evidence. Future well-conducted and clearly reported multicenter randomized controlled trials with a non-treatment control group are needed to provide the best scientific evidence relating to the effect of molar intrusion on the mandibular rotation and facial esthetic during open bite treatment.
